# Intermittent theta‐burst stimulation versus sham stimulation for suicidal ideation and psychiatric symptoms in depressive disorders: A systematic review and meta‐analysis of randomized controlled trials

**DOI:** 10.1002/pcn5.70427

**Published:** 2026-10-13

**Authors:** Muhammad Bilal, Muskan Kumari, Syeda Zainab Farooq, Fatima Sajjad, Meerab Sana Ullah, Muhammad Akif, Sana Iftikhar, Muhammad Nabeel Saddique, Mohammad Jalal Nazari

**Affiliations:** ^1^ Department of Psychiatry Wah Medical College Wah Cantt Punjab Pakistan; ^2^ Department of Psychiatry Liaquat University of Medical and Health Sciences (LUMHS) Jamshoro Pakistan; ^3^ College of Medicine Al Bukairiyah Sulaiman Al Rajhi University Qassim KSA Saudi Arabia; ^4^ Department of Psychiatry Foundation University Medical College Islamabad Pakistan; ^5^ Department of Psychiatry Sargodha Medical College Sargodha Pakistan; ^6^ Department of Psychiatry King Edward Medical University Lahore Pakistan; ^7^ Department of Psychiatry Shaikh Khalifa Bin Zayed Al Nahyan Medical and Dental College Lahore Pakistan; ^8^ Faculty of Medicine Kateb University Kabul Afghanistan

**Keywords:** anxiety, depression, intermittent theta‐burst stimulation, iTBS, sham–iTBS, sleep quality, suicidal ideation

## Abstract

Suicidal ideation, depressive symptoms, anxiety, and sleep disturbance remain difficult to manage in psychiatric populations, and intermittent theta‐burst stimulation (iTBS) has emerged as an accelerated, non‐invasive neuromodulation strategy for these outcomes. This meta‐analysis compares iTBS versus sham–iTBS across suicidal ideation, depression severity, anxiety severity, and sleep quality to guide clinical decision‐making. We systematically searched relevant electronic databases in accordance with Preferred Reporting Items for Systematic Reviews and Meta‐Analyses (PRISMA) guidelines. Continuous outcomes were pooled as standardized mean differences (SMDs) with 95% confidence intervals using random‐effects inverse‐variance models in RevMan 5.4. Heterogeneity was assessed using *I* ^2^ and *χ* ^2^ statistics, and leave‐one‐out sensitivity analyses were performed. A *p* value < 0.05 was considered statistically significant. Six studies were included. iTBS significantly reduced suicidal ideation versus sham–iTBS (SMD = −0.51; 95% CI: −0.93 to −0.09; *p* = 0.02; *I* ^2^ = 70%; *n* = 181 vs. 157), and the effect remained significant with lower heterogeneity after exclusion of Qin et al. (*p* = 0.02; *I* ^2^ = 29%). Depression severity improved with iTBS (SMD = −1.06; 95% CI: −1.64 to −0.48; *p* = 0.0003; *I* ^2^ = 80%; *n* = 162 vs. 142), with heterogeneity decreasing to 24% after exclusion of Huang et al. Anxiety severity also improved (SMD = −0.68; 95% CI: −1.27 to −0.09; *p* = 0.02; *I* ^2^ = 79%; *n* = 139 vs. 120), and heterogeneity decreased to 38% after exclusion of Huang et al. Sleep quality did not differ significantly between groups (SMD = −0.32; 95% CI: −0.98 to 0.34; *p* = 0.35; *I* ^2^ = 67%; *n* = 75 vs. 49), based on two trials. iTBS was associated with a reduction in suicidal ideation compared with sham stimulation, but the small evidence base and between‐study heterogeneity limit certainty. Secondary analyses suggested improvements in depression and anxiety severity, whereas sleep quality did not differ between groups. Larger, standardized randomized controlled trials with longer follow‐up are needed to determine the magnitude and durability of any antisuicidal effect.

## INTRODUCTION

Depressive disorders represent a major global public health challenge, affecting approximately 332 million people worldwide in 2021 and contributing to more than 56 million disability‐adjusted life years, with women experiencing a disproportionately greater burden.^1^ Among the most serious clinical consequences of depressive illness is suicidal ideation, a complex and heterogeneous phenomenon due to clinical, psychological, social, and biological factors.^2^ The substantial global burden of suicide, which accounted for approximately 800,000 deaths worldwide, while a history of suicide attempts remains an important predictor of both recurrent attempts and completed suicide.^3^

Antidepressants remain the primary pharmacological treatment for depression, with selective serotonin reuptake inhibitors (SSRIs), serotonin and norepinephrine reuptake inhibitors (SNRIs), tricyclic antidepressants (TCAs), monoamine oxidase inhibitors (MAOIs), and newer agents widely used across healthcare settings. Their use has increased globally, particularly in high‐income countries, largely due to greater prescribing of SSRIs and newer antidepressants.^4^, ^5^ A major limitation of conventional antidepressant therapy is the delayed and variable onset of clinical benefit. Although early symptom improvement within 2–4 weeks may predict remission by 8–12 weeks, the absence of an early response often indicates a lower likelihood of favorable long‐term outcomes.^6^ Despite their central role in treating anxiety and depressive disorders, SSRIs and SNRIs are associated with early‐emerging adverse effects, including activation, gastrointestinal symptoms, sedation, and sleep disturbances, as well as later effects such as weight gain. Although several symptoms may diminish during treatment, tolerability concerns can impair adherence and lead to treatment discontinuation.^7^

Repetitive transcranial magnetic stimulation (rTMS) is a non‐invasive neuromodulation technique that delivers targeted magnetic pulses to cortical regions, with established therapeutic efficacy in selected neurological and psychiatric conditions.^8^ Intermittent theta‐burst stimulation (iTBS) is a patterned form of repetitive TMS that may alleviate treatment‐resistant depression by restoring disrupted synaptic plasticity in the prefrontal cortex.^9^ Clinically, iTBS can be delivered in approximately 3 min compared with 37.5 min for standard 10‐Hz rTMS, while the THREE‐D trial demonstrated that iTBS was non‐inferior to 10‐Hz rTMS in reducing depressive symptoms among adults with treatment‐resistant depression.^10^ Accelerated protocols that deliver multiple sessions per day, often with functional‐connectivity‐guided targeting, compress a full treatment course into days and have produced high remission rates in treatment‐resistant depression.^11^

Evidence regarding the antisuicidal effects of iTBS remains mixed. In antidepressant‐free adults with treatment‐resistant depression, a four‐day accelerated iTBS protocol was associated with a sustained reduction in suicide risk; however, this improvement was not specific to active stimulation and was also independent of antidepressant response.^12^ Recently randomized sham‐controlled studies have subsequently examined iTBS in adults and adolescents with treatment‐resistant depression, suicide attempts, suicidal ideation, anhedonia, and non‐suicidal self‐injury.^13^, ^14^ Evidence from randomized sham‐controlled trials specifically evaluating suicidal ideation as a primary outcome remains limited, while the effects of iTBS on clinically related symptom domains, particularly anxiety and sleep quality, have not yet been comprehensively quantified.

Accordingly, we performed a systematic review and meta‐analysis of randomized controlled trials comparing iTBS with sham stimulation in patients with depressive disorders. The primary objective was to estimate the effect of iTBS on suicidal ideation. Secondary objectives were to evaluate depression severity, anxiety severity, sleep quality, and the completeness of reported safety outcomes.

## METHODOLOGY

This review was conducted in accordance with the Cochrane Handbook for Systematic Reviews of Interventions and reported using the Preferred Reporting Items for Systematic Reviews and Meta‐Analyses (PRISMA) 2020 statement.^15^ This systematic review and meta‐analysis were registered with the International Prospective Register of Systematic Reviews CRD420261466294.^16^

### Information sources and search strategy

PubMed, Scopus, EMBASE databases, and ClinicalTrials.gov were searched from inception to July 2026 by two independent reviewers. The search strategy combined controlled vocabulary and entry terms related to depressive disorders, suicidal ideation or self‐harm, iTBS, and sham stimulation like ((((((((((((((((“Depressive Disorder”[Mesh]) OR (Depressive Disorders)) OR (Neurosis, Depressive)) OR (Depression, Endogenous)) OR (Unipolar Depression)) OR (Depressive Syndrome)) OR (“Major Depressive Disorder”[Mesh]) (Depressive Disorders, Major)) OR (Disorder, Major Depressive)) OR (Major Depressive Disorders)) OR (Psychosis, Involutional)) OR (“Depressive Disorder, Treatment‐Resistant”[Mesh]) OR (Depressive Disorder, Treatment Resistant)) OR (Disorder, Treatment‐Resistant Depressive)) OR (Resistant Depressions, Treatment)) OR (Therapy Resistant Depression)) AND (Intermittent Theta Burst Stimulation)) OR (iTBS)). Full database‐specific search strategies provided in Table S1.

### Study selection criteria

Studies were eligible when they: (1) used a randomized controlled design; (2) enrolled adolescents or adults with MDD, treatment‐resistant depression, or a depressive disorder accompanied by suicidal ideation, suicide attempt, or non‐suicidal self‐injury; (3) evaluated active iTBS, including conventional or accelerated schedules; (4) used a sham–iTBS or sham transcranial magnetic stimulation comparator; (5) reported extractable continuous data for suicidal ideation, depression severity, anxiety severity, sleep quality, or treatment safety; and (6) were available as full peer‐reviewed reports. No restriction was placed on stimulation dose, targeting strategy, concomitant stable treatment, or follow‐up duration.

Non‐randomized studies, uncontrolled case series, conference abstracts without sufficient data, reviews, animal studies, and trials of continuous theta‐burst stimulation, conventional rTMS, deep transcranial magnetic stimulation, or electroconvulsive therapy without a separately reportable iTBS arm were excluded. Studies without a sham comparator or without data suitable for quantitative extraction were also excluded. In Figure 1, the category “Non‐randomized controlled trial (RCT) or uncontrolled design” denotes reports that lacked randomized allocation or a concurrently randomized sham comparator; this category included non‐randomized comparative studies, uncontrolled single‐arm studies, case series, study protocols, reviews, and conference reports without sufficient randomized data.

**Figure 1:**
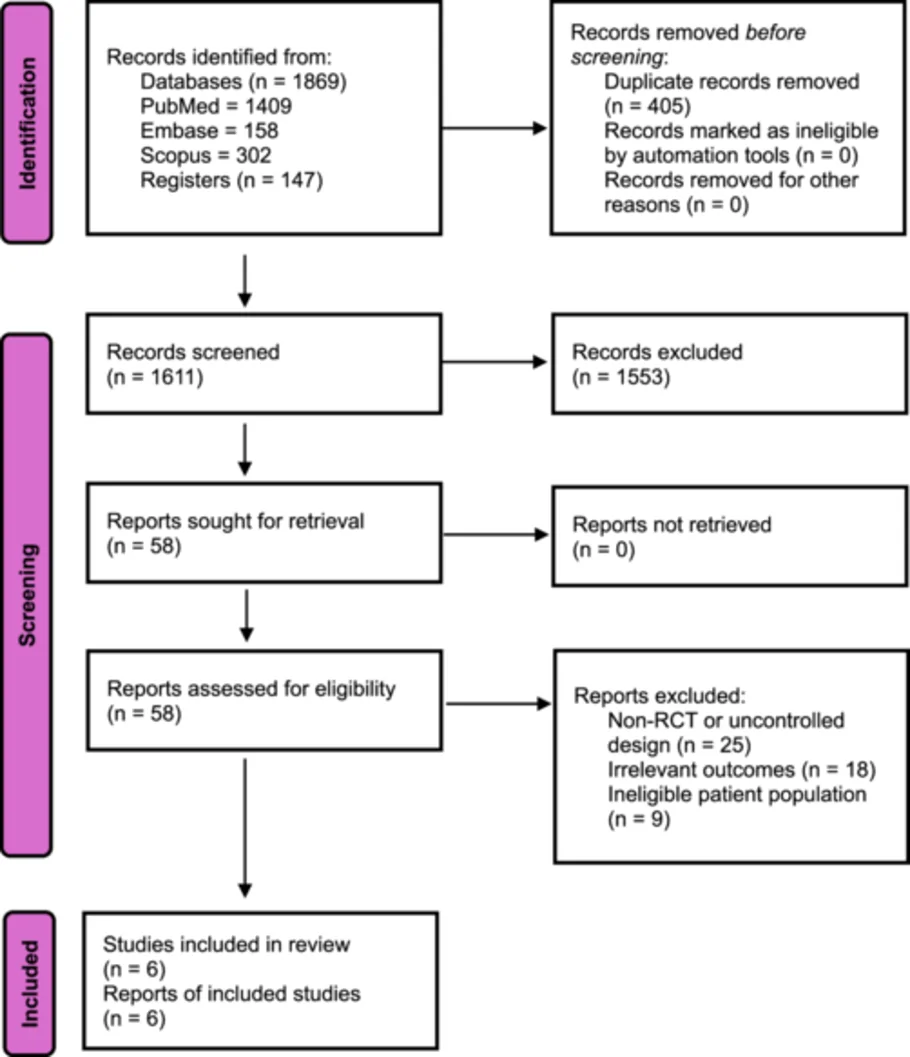
Preferred Reporting Items for Systematic Reviews and Meta‐Analyses (PRISMA) flow diagram.

### Study selection process

Records were managed and screened using web‐based tool Rayyan.^17^ Duplicate records were removed by one reviewer. Titles and abstracts were screened independently by two independent reviewers and all conflicts were resolved by third reviewer. Potentially eligible full texts were assessed independently, and exclusion reasons were recorded. Disagreements were resolved by the third reviewer.

### Data extraction and quality assessment

Data extraction was performed by two independent authors from include studies using standardized google sheet and all discrepancies resolved by third author through discussion. The data extraction sheet included publication year, country, trial registration, study design, population, sample size, participant baseline characteristics, targeting method, stimulation device and intensity, burst frequency, pulses per session, sessions per day, total sessions, treatment duration, follow‐up, outcome instrument, and safety reporting. The primary outcome was suicidal ideation measured with any validated scale, including the Beck Scale for Suicide Ideation (BSSI) and related versions. Secondary outcomes were depression severity, anxiety severity, and sleep quality. Adverse events were planned as a secondary safety outcome, but reporting was too sparse and inconsistent to support a reliable pooled analysis.

Two reviewers were assigned to assess each randomized trial with the Cochrane Risk of Bias 2 (RoB 2) tool independently,^18^ covering the randomization process, deviations from intended interventions, missing outcome data, outcome measurement, and selection of the reported result.^18^ Disagreements were resolved through discussion.

### Statistical analysis

All statistical analyses were performed using Review Manager (RevMan), version 5.4 (Cochrane Collaboration). Continuous outcomes, such as suicidal ideation, depression severity, anxiety severity and sleep quality were pooled using standardized mean difference (SMD) with 95% CI, a random‐effects model based on the DerSimonian–Laird method was used throughout the analyses. Statistical heterogeneity was assessed using the Cochrane chi‐squared (*Q*) test, and quantified using the *I* ^2^ statistic. *I* ^2^ values of 25%, 50%, and 75% were interpreted as low, moderate, and substantial heterogeneity, respectively. When substantial heterogeneity was observed, leave‐one‐out sensitivity analyses were performed by removing each study sequentially to evaluate its effect on the pooled estimates, heterogeneity, and statistical significance. In addition, all analyses were repeated using a random‐effect model to examine the robustness of the findings. For all outcomes analysis, *p* value below 0.05 was considered statistically significant.

## RESULTS

### Study selection

The search identified 2016 records: 1409 from PubMed, 302 from Scopus, 158 from Embase, and 147 from ClinicalTrials.gov. After removal of 405 duplicates, 1611 records underwent title and abstract screening. Of these, 1553 were excluded, leaving 58 reports assessed for eligibility. Fifty‐two reports were excluded: 25 had a non‐RCT or uncontrolled design, 18 reported irrelevant outcomes, and 9 enrolled an ineligible patient population. Six randomized controlled trials were included in the qualitative and quantitative syntheses.^12^, ^13^, ^14^, ^19^, ^20^, ^21^ Figure 1 presents the complete PRISMA study‐selection process.

### Study characteristics

The six trials randomized 352 participants and were conducted in Belgium, China, and Taiwan between 2016 and 2026. Populations ranged from adolescents with MDD and suicide attempt or non‐suicidal self‐injury to adults with MDD, anhedonia, suicidal ideation, or treatment‐resistant depression. All trials stimulated the left dorsolateral prefrontal cortex (DLPFC), but targeting ranged from the conventional 5‐cm or F3 approach to individualized functional magnetic resonance imaging‐guided reward‐circuit targeting. Pulse dose varied from 600 to 1800 pulses per session, treatment frequency ranged from one to five sessions per day, and total exposure ranged from 10 to 30 sessions. Follow‐up ranged from the end of treatment to 6 weeks. Table 1 summarizes the parameters of included studies.

**Table 1:** Stimulation parameters of included studies.

Author (year)	Intervention	Stimulation location	Targeting method	Intensity (% MT)	Frequency (Hz)	Pulses/sess	Sess/day	Intersession interval	Treatment days	Total sessions
Desmyter et al. (2016)	iTBS	L‐DLPFC	Magstim Rapid2 Plus1 system (localization method NR)	110% MT	50 Hz	1620	5 sessions	15 min	4 days	20 sessions
Zhao et al. (2023)	Active iTBS	L‐DLPFC	Standard 5‐cm method	80% AMT	50‐Hz burstsat 5 Hz	1800	1 session	N/A	10 weekdays over 2 weeks	10 sessions
Huang et al. (2025)	iTBS	L‐DLPFC‐NAcc circuit	Individualized fMRI‐guided neuronavigation (Brainsight)	80% RMT	50‐Hz burstsat 5 Hz	600	1 session	N/A	15 days	15 sessions
Qin et al. (2025)	iTBS	L‐DLPFC	MagPro X100/B65 coil (localization method NR)	80% RMT	5‐Hz bursts	1800	5 sessions	60 min	5 consecutive days	25 sessions
Pu et al. (2026)	iTBS + stable medication	L‐DLPFC	International 10–20 EEG system (F3 site)	90% RMT	50‐Hz burstsat 5 Hz	1800	2 sessions	Approx. 50 min	10 days	20 sessions
Chen et al. (2026)	iTBS	L‐DLPFC	Magstim D70 alpha figure‐8 coil (localization method NR)	85.5% MT	3‐pulse 50‐Hz burstsevery 200 ms	1800	3 sessions	60 min	10 days (over 2 weeks)	30 sessions

Abbreviations: AMT, active motor threshold; DLPFC, dorsolateral prefrontal cortex; EEG, electroencephalography; fMRI, functional magnetic resonance imaging; Hz, hertz; iTBS, intermittent theta‐burst stimulation; L, left; MT, motor threshold; NAcc, nucleus accumbens; NR, not reported; RMT, resting motor threshold.

### Risk of bias

Assessment using the Cochrane RoB 2 tool indicated generally favorable methodological quality across the included randomized controlled trials. Three trials were judged to have a low overall risk of bias, while three raised some concerns; none were rated as having a high risk of bias. Concerns were primarily related to the randomization process and missing outcome data, whereas the remaining domains were generally assessed as low risk. The risk‐of‐bias summary and traffic‐light plots are presented in Figure S2.

### Suicidal ideation

Six studies involving 338 participants (181 iTBS group: 157 sham–iTBS group) evaluated suicidal ideation. The pooled analysis demonstrated a statistically significant reduction in suicidal ideation among participants receiving iTBS compared with sham stimulation (SMD = −0.51; 95% CI: −0.93 to −0.09; *p* = 0.02). Moderate to substantial heterogeneity was observed across studies (*I* ^2^ = 70%). The corresponding forest plot is presented in Figure 2. Leave‐one‐out sensitivity analysis showed that the removal of Qin et al. (2025) reduced heterogeneity from 70% to 30% and the pooled result remained statistically significant Figure S3.

**Figure 2:**
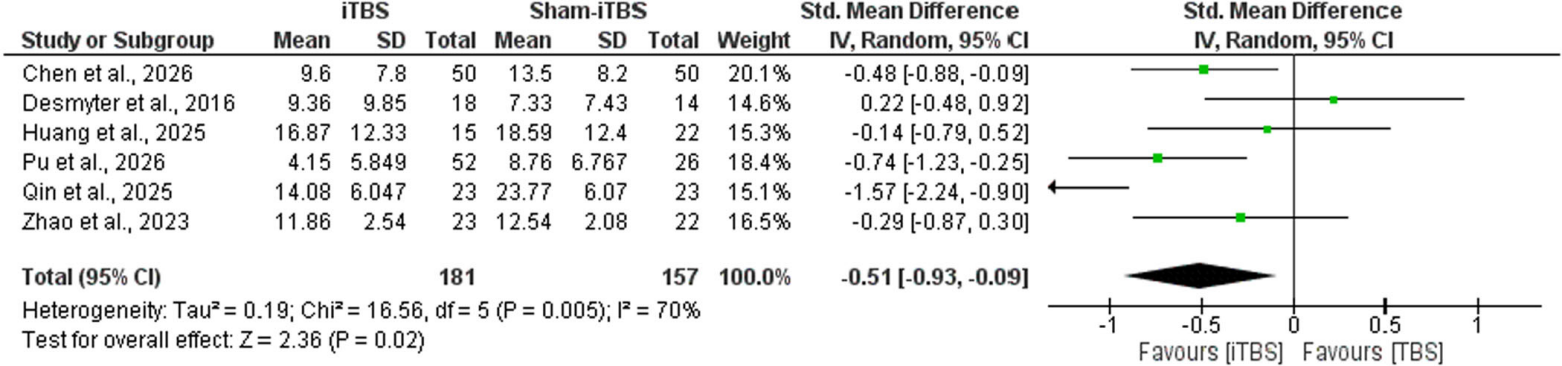
Forest plot of suicidal ideation. CI, confidence interval; iTBS, intermittent theta‐burst stimulation.

### Depression severity

Five studies involving 304 participants (162 in the iTBS group and 142 in the sham–iTBS group) evaluated depression severity. The pooled analysis favored iTBS (SMD = −1.06; 95% CI: −1.64 to −0.48; *p* = 0.0003), with considerable heterogeneity (*I* ^2^ = 80%) (Figure 3). In the leave‐one‐out sensitivity analysis, exclusion of Huang et al. reduced heterogeneity from 80% to 24%, and the pooled result continued to favor iTBS (Figure S4).

**Figure 3:**
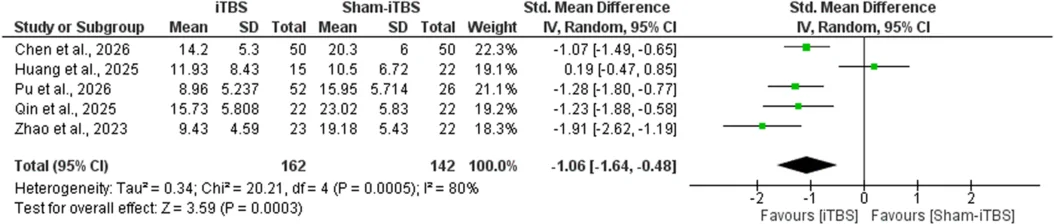
Forest plot of depression severity. CI, confidence interval; iTBS, intermittent theta‐burst stimulation.

### Anxiety severity

Four trials contributed 259 participants (139 in the iTBS group and 120 in the sham–iTBS group). The pooled effect favored iTBS (SMD = −0.68; 95% CI: −1.27 to −0.09; *p* = 0.02), with high heterogeneity (*I* ^2^ = 79%) (Figure 4). Exclusion of Huang et al. produced a larger pooled effect (SMD = −0.95; 95% CI: −1.72 to −0.17; *p* = 0.034) and reduced heterogeneity to 38% (Figure S5).

**Figure 4:**
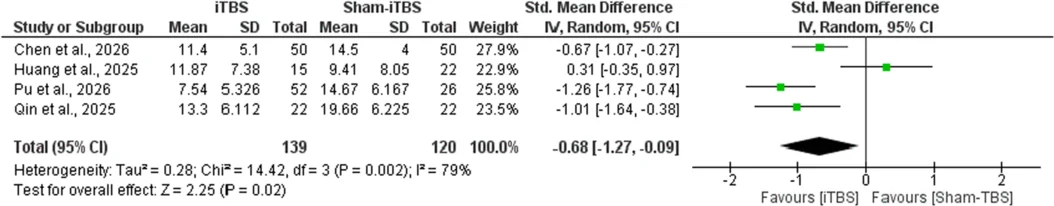
Forest plot of anxiety severity. CI, confidence interval; iTBS, intermittent theta‐burst stimulation.

### Sleep quality

Two studies involving 124 participants (75 iTBS group; 49 sham–iTBS group) evaluated sleep quality. The pooled analysis showed no statistically significant difference in sleep quality between the iTBS and sham stimulation groups (SMD = −0.32; 95% CI: −0.98 to 0.34; *p* = 0.35). Substantial heterogeneity was observed among the included studies (*I* ^2^ = 67%);. The corresponding forest plot is presented in Figure 5.

**Figure 5:**
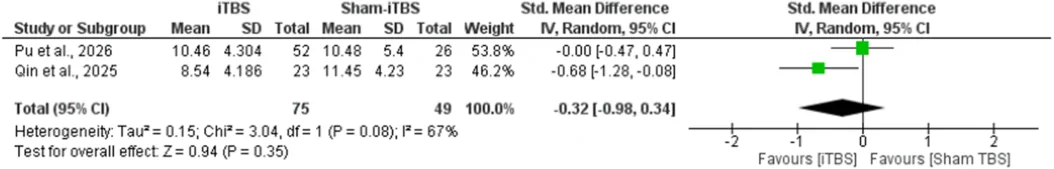
Forest plot of sleep quality. CI, confidence interval; iTBS, intermittent theta‐burst stimulation.

## DISCUSSION

In this meta‐analysis of six randomized, sham‐controlled trials, active iTBS was associated with a modest reduction in suicidal ideation, the primary outcome, compared with sham stimulation. The association remained statistically significant in the leave‐one‐out sensitivity analysis, although the effect magnitude varied and between‐study heterogeneity was substantial. Findings for secondary outcomes like depression severity, anxiety severity were significantly improved except for sleep quality which shows no improvement, although this analysis included only a small number of trials.

The six trials differed in population, stimulation schedule, targeting method, and timing of outcome assessment. Desmyter et al. evaluated a 4‐day accelerated crossover protocol in antidepressant‐free adults with treatment‐resistant depression; suicide‐risk scores decreased over time, but the reduction was not specific to active stimulation.^12^ Zhao et al. found that 10 daily sessions over 2 weeks reduced suicidal ideation in adolescents with major depressive disorder and a previous suicide attempt, with larger improvements among those with more severe baseline symptoms.^19^ Huang et al. used individualized left DLPFC‐nucleus accumbens targeting and found greater improvement in worst‐point suicidal ideation after iTBS than after sham, although the past‐week measure did not show a significant group‐by‐time interaction.^20^ Qin et al. studied an accelerated 5‐day protocol in adolescents with major depressive disorder and non‐suicidal self‐injury and reported greater reductions in self‐injury severity and ideation at 4 weeks with active iTBS.^14^ Pu et al. found that a 10‐day adjunctive iTBS course produced a greater post‐treatment reduction in BSSI scores than sham while medications remained stable.^13^ Chen et al. enrolled 100 inpatients with treatment‐resistant depression and suicidal ideation; active accelerated iTBS separated from sham by Week 2, yielded higher response rates, and maintained benefit through Week 6.^21^ The direct enrollment of patients with suicidal symptoms is clinically important because the THREE‐D trial excluded active suicidal intent,^10^ and consensus recommendations have historically advised considering established rapid‐acting treatments, such as electroconvulsive therapy, for patients with acute suicidal ideation.^22^ The broadly favorable findings in several parallel‐group trials contrast with the nonspecific improvement in the early crossover trial and may partly explain the observed heterogeneity. Nevertheless, the small samples and varied outcome instruments preclude firm conclusions about which protocol or patient subgroup benefits most.

The reduction in suicidal ideation was the most clinically important finding, as suicidal ideation predicts future suicidal behavior and often responds slowly or inconsistently to conventional antidepressant treatment. Further variation may reflect differences in the suicidal outcomes studied and the assessment tools used. These findings suggest that the antisuicidal effects of iTBS may not be entirely mediated by its antidepressant effects. The substantial heterogeneity in the suicidal‐ideation analysis likely reflects differences in age, baseline suicidality, depressive subtype, treatment history, stimulation intensity, pulse dose, sessions per day, total sessions, target localization, follow‐up, and the construct measured (suicidal ideation, suicide risk, or self‐injury‐related ideation).^23^ These differences limit the interpretability of a single pooled effect and support the use of standardized suicidality measures and stimulation protocols in future trials.

The observed reduction in depressive symptoms aligns with the broader theta‐burst stimulation literature, where meta‐analyses have demonstrated a moderate benefit compared with sham treatment.^24^ Previous meta‐analyses have consistently demonstrated that theta‐burst stimulation is effective in improving depressive symptoms compared with sham stimulation, supporting its role as a promising non‐invasive neuromodulation therapy for major depressive disorder.^25^ Earlier studies assessed the efficacy of different theta‐burst protocols, including intermittent, continuous, and bilateral stimulation. while more recent studies have further supported the efficacy of accelerated iTBS in the treatment of major depressive episodes.^23^ Although iTBS demonstrated significant benefits for suicidal ideation, depression severity, and anxiety symptoms, considerable heterogeneity was observed across several outcomes, indicating variability in treatment effects among the included studies, differences in targeting approaches, ranging from conventional anatomical methods to individualized neuronavigation, may have contributed to variability across studies. Such heterogeneity was also noted in other meta‐analyses of TBS, indicating that variability between studies is a recurring challenge in this field.

Our findings are consistent with these reports, demonstrating significant improvements in depression severity following iTBS. Importantly, our meta‐analysis extends the current evidence by pooling and analyzing outcomes beyond depression, including suicidal ideation, anxiety severity, sleep quality, and safety. The observed reduction in suicidal ideation is particularly noteworthy given the clinical relevance of this outcome in patients with depression. These findings suggest that iTBS may represent a useful adjunctive option for patients with depression and persistent suicidal symptoms, particularly when conventional treatments have not provided sufficient improvement. The main contribution of this review is the quantitative synthesis of sham‐controlled iTBS evidence for suicidal ideation. The pooled estimate suggests a possible adjunctive benefit, but it should not be interpreted as support for replacing established acute suicide‐risk management or as evidence that iTBS prevents suicide attempts or suicide deaths. A strength of this meta‐analysis is its restriction to randomized, sham‐controlled trials and its inclusion of recently published studies that directly enrolled patients with suicidal or self‐injurious symptoms. Adverse‐event reporting, however, remained too sparse and inconsistent to support a pooled safety estimate.

### Limitations

This meta‐analysis has several limitations. First, only six randomized controlled trials were available, and most had small samples. Second, populations, stimulation protocols, follow‐up durations, and outcome constructs differed, and not all trials were primarily designed to evaluate suicidal ideation. Third, the pooled outcome combined instruments assessing suicidal ideation, suicide risk, or self‐injury‐related ideation, which may not represent identical constructs. Finally, adverse events were reported too sparsely and inconsistently for a reliable quantitative assessment of tolerability. These limitations reduce the precision and generalizability of the pooled estimate.

## CONCLUSION

Overall, the available evidence suggests that iTBS may reduce suicidal ideation when used as an adjunct to standard treatment for depressive disorders. This conclusion remains provisional because only six small trials were available, protocols and outcome constructs varied, and follow‐up was generally short. The findings do not establish effects on suicide attempts, suicide mortality, or long‐term recurrence. Larger multicenter randomized controlled trials should use standardized suicidality measures, prespecified safety reporting, and longer follow‐up to determine the magnitude, durability, and clinical relevance of any antisuicidal effect.

## USE OF ARTIFICIAL INTELLIGENCE

No use of AI.

## AUTHOR CONTRIBUTIONS

**Muhammad Bilal**: Conceptualization; methodology; data curation; writing—original draft preparation; writing—reviewing and editing. **Muskan Kumari**: Methodology; writing—original draft preparation; writing—reviewing and editing. **Syeda Zainab Farooq**: Writing—original draft preparation; Writing—reviewing and editing. **Fatima Sajjad**: Writing—original draft preparation; writing—reviewing and editing. **Meerab Sana Ullah**: Writing—original draft preparation; writing—reviewing and editing. **Muhammad Akif**: Writing—original draft preparation; writing—reviewing and editing. **Sana Iftikhar**: Writing—original draft preparation; writing—reviewing and editing. **Muhammad Nabeel Saddique**: Writing—original draft preparation; writing—reviewing and editing. **Mohammad Jalal Nazari**: Writing—original draft preparation; writing—reviewing and editing.

## CONFLICT OF INTEREST STATEMENT

The authors declare no conflicts of interest.

## ETHICS APPROVAL STATEMENT

N/A.

## PATIENT CONSENT STATEMENT

N/A.

## CLINICAL TRIAL REGISTRATION

N/A.

## Supporting information

Supporting File

## Data Availability

All data used in this systematic review and meta‐analysis are provided in the manuscript and supplementary files. No new datasets were generated.
