# Supplementary material for: Intermittent theta‐burst stimulation versus sham stimulation for suicidal ideation and psychiatric symptoms in depressive disorders: A systematic review and meta‐analysis of randomized controlled trials
Source: PCN Rep. 2026 Oct 13;5(4):e70427. doi: 10.1002/pcn5.70427 (PMC13660502; doi:10.1002/pcn5.70427)
Supplement: Supplementary file 1 [file PCN5-5-e70427-s001.docx]

**Supplementary File**

**Search Strategy**

| **PubMed** | **20-6-2026,** | ((((((((((((((("Depressive Disorder"[Mesh]) OR (Depressive Disorders)) OR (Neurosis, Depressive)) OR (Depression, Endogenous)) OR (Unipolar Depression)) OR (Depressive Syndrome)) OR ("Major Depressive Disorder"[Mesh]) (Depressive Disorders, Major)) OR (Disorder, Major Depressive)) OR (Major Depressive Disorders)) OR (Psychosis, Involutional)) OR ("Depressive Disorder, Treatment-Resistant"[Mesh]) OR (Depressive Disorder, Treatment Resistant)) OR (Disorder, Treatment-Resistant Depressive)) OR (Resistant Depressions, Treatment)) OR (Therapy Resistant Depression)) AND (Intermittent Theta Burst Stimulation)) OR (iTBS) | **1409** |
| --- | --- | --- | --- |
| **Embase** | **20-6-2026** | ('depression'/exp OR 'major depression'/exp OR depression OR 'depressive disorder' OR mdd OR 'treatment resistant depression' OR trd) AND ('theta burst stimulation' OR 'intermittent theta burst stimulation' OR itbs OR tbs OR rtms OR 'transcranial magnetic stimulation') AND (suicide OR suicidality OR 'suicidal ideation' OR 'suicide attempt' OR 'self harm' OR nssi OR 'non suicidal self injury') AND (randomized OR randomised OR placebo OR sham) | **158** |
| **Scopus** | **20-06-2026** | ( TITLE-ABS-KEY ( depressive disorders ) AND TITLE-ABS-KEY ( Intermittent Theta Burst Stimulation ) ) | **302** |
| **ClinicalTrials.gov** | **19-06-2026** | Condition/disease: Depressive Disorders  Intervention/treatment: Intermittent Theta Burst Stimulation | **147** |

**Supplementary Table S1.** Complete search strategies for all databases

**Supplementary Figure S2: (A). Traffic Plot (B). Summary Plot**

**Supplementary Figure S3: Sensitivity Analysis of Suicidal Ideation**

**Supplementary Figure S4: Sensitivity Analysis of Depression Severity**

**Supplementary Figure S5: Sensitivity Analysis of Anxiety severity**
